# Metabolomics identifies plasma biomarkers of localized radiation injury

**DOI:** 10.1038/s41598-025-85717-5

**Published:** 2025-01-16

**Authors:** Lucie Ancel, Stéphane Grison, Olivier Gabillot, Jules Gueguen, Ljubica Svilar, Bernard Le Guen, Gaëtan Gruel, Marc Benderitter, Jean-Charles Martin, Maâmar Souidi, Radia Tamarat, Stéphane Flamant, Mohamed Amine Benadjaoud

**Affiliations:** 1 Institut de Radioprotection et de Sureté Nucléaire (IRSN), PSE-SANTE/SERAMED/LRAcc, 31 av de la Division Leclerc, Fontenay-aux-Roses, 92260 France; 2 Institut de Radioprotection et de Sureté Nucléaire (IRSN), PSE-SANTE/SESANE/LRTox, Fontenay-aux-Roses, 92260 France; 3 Centre Cardiovasculaire et Nutrition (C2VN), CRIBIOM, Aix Marseille Université, Marseille, 13007 France; 4 Électricité de France (EDF), DPN, 1 place Pleyel, Saint Denis, 93382 France; 5 Institut de Radioprotection et de Sureté Nucléaire (IRSN), PSE-SANTE/SERAMED, Fontenay-aux-Roses, 92260 France; 6 Institut de Radioprotection et de Sureté Nucléaire (IRSN), PSE-SANTE, Fontenay-aux-Roses, 92260 France; 7 C2VN, INRAE, INSERM, BIOMET, Aix Marseille Université, Marseille, 13007 France

**Keywords:** Metabolite, Ionizing radiation, Molecular biomarkers, Localized radiation injury, Metabolomics, Diagnostic markers

## Abstract

A radiological accident may result in the development of a local skin radiation injury (LRI) which may evolve, depending on the dose, from dry desquamation to deep ulceration and necrosis through unpredictable inflammatory waves. Therefore, early diagnosis of victims of LRI is crucial for improving medical care efficiency. This preclinical study aims to identify circulating metabolites as biomarkers associated with LRI using a C57BL/6J mouse model of hind limb irradiation. More precisely, two independent mice cohorts were used to conduct a broad-spectrum profiling study followed by a suspect screening analysis performed on plasma metabolites by mass spectrometry. An integrative analysis was conducted through a multi-block sparse partial least square discriminant analysis (sPLS-DA) to establish multi-scale correlations between specific metabolites levels and biological, physiological (injury severity), and functional parameters (skin perfusion). The identified biomarker signature consists in a 6-metabolite panel including putrescine, uracil, 2,3-dihydroxybenzoate, 3-hydroxybenzoate, L-alanine and pyroglutamate, that can discriminate mice according to radiation dose and injury severity. Our results demonstrate relevant molecular signature associated with LRI in mice and support the use of plasma metabolites as suitable molecular biomarkers for LRI prognosis and diagnosis.

**Supplementary Information:**

The online version contains supplementary material available at 10.1038/s41598-025-85717-5.

## Introduction

Local exposure to high dose of ionizing radiation (IR) may arise as a consequence of industrial or medical accidents^1^. In addition, the ever-growing threat of malevolent acts or terrorist attacks involving radiological exposure devices, in regard to the current geopolitical situation, increases the risk of mass casualties experiencing radiation induced syndromes including cutaneous radiation injuries. In the situation of a radiological emergency involving a large number of individuals, the medical management of the victims is of primary importance.

Localized skin radiation injury (LRI) resulting from acute local irradiation or external contamination may extend over time into underlying muscle and bone tissues. The severity of these lesions depends on the received dose and the volume of tissue exposed (skin, muscle, bone). In addition, LRI is characterized by the development of a dynamic injury, evolving in recurrent waves of inflammation, which can be challenging to predict^2,3^. Erythema or edema may appear within a few hours following IR exposure. After a period of latency with absence of obvious clinical signs and variable duration depending on the received dose and irradiated volume, a dry, then moist desquamation, ulceration and ultimately tissue necrosis may occur for doses > 20–25 Gy^4^. Late chronic effects may occur months to years from time of exposure, which typically manifest as telangiectasia, dermal fibrosis, microvascular damage, skin atrophy and ulcers^2,4^. As a continuously renewing organ containing highly proliferative stem cells, the skin is particularly radiosensitive. IR exposure of the skin induces destruction of basal keratinocytes and hair follicle stem cells, associated with massive production of free radicals, and inflammatory cell infiltration^2,4^.

Current diagnostic approaches include clinical assessment, magnetic resonance imagery and plasma CRP quantification, but are usually performed after first manifestations of symptoms^5^. Treatment recommendations for severe cases involve a dosimetry-guided excision surgery combined with skin autograft and cell therapy as an adjuvant treatment which seems to be more effective with early implementation^3^. Therefore, optimal medical care of patients with LRI would benefit from identification of early relevant biomarkers of injury severity, especially in the situation of radiological emergency consecutive to mass accident. Such early identification of potential victims of severe LRI would allow victim surveillance, correct assignment to appropriate hospitals, and patient preparation for adapted therapeutic procedures.

In this regard, a limited number of circulating molecular markers have been described with diagnostic or prognostic value for radiation induced organ injury, including Flt3-ligand^6,7^ or citrulline^8,9^ for bone marrow and gastrointestinal damage, respectively. In contrast, there is currently no operational molecular marker for diagnosis or prognosis assistance of the LRI. Nonetheless, preclinical studies using a mouse model of skin radiation injury have identified early serum proteomic signatures associated with injury grade, setting the basis of using systemic biomarkers for diagnosis or prognosis of localized IR exposure^10,11^. Moreover, we recently identified a diagnostic microRNA (miRNA) signature of localized radiation injury in mice plasma, with a panel of 8 miRNAs able to discriminate animals according to their dose group and injury severity^12^.

Several preclinical studies have previously explored the interest of omics biomarkers, including metabolites, miRNA, and gene expression, for the early identification of individuals exposed to total body irradiation^13–15^, or in the context of chronic contamination with cesium-137 or uranium^16–18^. Indeed, the development of omics technologies, and in particular metabolomics, offers unprecedented insights into the characterization of the health status of a patient or the identification of dysfunctional biological processes^15,19^. Applying such a global approach on biological samples collected by minimally invasive means would allow to obtain molecular signatures with diagnostic or prognostic value. In this regard, a number of preclinical studies conducted on rodents and non-human primates (NHP) have established metabolomic signatures in blood and urine in response to total body irradiation (TBI)^20–22^. While these studies were essentially oriented towards the improvement of biological dosimetry assessment, metabolomic signatures may contribute to develop novel diagnostic biomarkers of radiation exposure, as well as prognostic molecular markers of injury evolution and severity. In addition, a set of metabolites was identified as commonly perturbed in biological fluids consecutive to IR exposure in several animal and human studies^15^. Among them, the most frequently perturbed metabolites which displayed highest consistency in directional trend included citrulline, citric acid, creatine, and uric acid, making them good diagnostic candidate markers. Of note, the large majority of the studies involved TBI settings. Interestingly, metabolomics analysis was used to decipher the molecular changes occurring in irradiated skin tissue using a pig model of cutaneous radiation injury^23^. Changes were observed in a time and dose-dependent manner in a large number of metabolites associated with oxidative stress, lipid metabolism, protein degradation, and vascular damage.

However, performing tissue biopsies for diagnosis assistance in the context of human cutaneous radiation injury is not recommended, since excision or surgery of irradiated tissues trigger the inflammatory waves and lead to extension of the injury^3,24^. Therefore, in order to identify minimally invasive and non-detrimental molecular markers associated with radiation burn, we designed metabolomics profiling studies of biological fluids using an experimental mouse model of local IR exposure. Here, we report the results of the pilot study aiming at characterizing blood metabolome of mice locally exposed to different doses of IR, resulting in radiation burns of different severity grades. We found that mice plasma displayed metabolite signatures allowing to segregate irradiated animals from non-exposed littermates. A specific combination of metabolites related to inflammation, oxidative stress, and skin damage allowed the discrimination of irradiated mice according to radiation dose and injury severity.

## Material & methods

### Animals and experimental procedure

Two independent cohorts of sixty male, 8-week-old C57/BL6 mice (Janvier Labs, Le Genest Sainte Isle, France) were used in this study. Housing conditions and irradiation procedure were conducted as previously described^12^. Briefly, mice were irradiated under gas anesthesia on their hindlimb using a medical linear accelerator (Elekta Synergy^®^, Elekta SAS, Boulogne-Billancourt, France) delivering 10 MV X-rays (mean photon energy of 1.4 MeV) at doses of 0, 20, 40 and 80 Gy (*N* = 15/group for each animal cohort) in order to induce varying degrees of lesion severity with slight (20 Gy), moderate (40 Gy) to severe (80 Gy) injuries^12^. A control group of animals (*N* = 15 for each cohort) was sham irradiated using the same conditions of anaesthesia and manipulation. Every manipulation handling and non-invasive measurements on animals were performed under anesthesia with isoflurane. Mice were euthanized 14 days after irradiation with intracardiac blood sampling followed by cervical dislocation. One cohort of animals (*n* = 60) was used for broad-spectrum study, and the other one (*n* = 60) for suspect screening analysis (SSA). During the experiment, 5 mice from the 40 Gy group of the SSA study were excluded due to aggressive behavior among littermates.

### Ethic approvals

Experiments were conducted in compliance with French veterinary guidelines and those formulated by the European Community for experimental animal use, and protocols were approved by the institutional animal experimentation and ethics committee (protocol P19-17, agreement no. 22393-2019101116207862v1) and were carried out in accordance with the ARRIVE guidelines.

### Plasma collection

Blood was collected before euthanasia 14 days after irradiation and processed into platelet-poor plasma (PPP) following the procedure described in Ancel et al.^12^. PPP samples were kept frozen at -80 °C until further use for molecular biology analysis. Due to technical and experimental constraints, only plasma samples with sufficient volume were used for molecular analysis.

### Clinical and biochemical analyses

The evolution of radiation injury was monitored at day 7, 10 and 14 after irradiation with a semi-quantitative injury score established for each mouse^12^. Skin blood perfusion was analysed at those time points using a Moor LDI2 Laser Doppler Imager (Moor Instruments ltd, Axminster, UK). Animal weight was monitored at day 0, 7 and 14 post-irradiation.

### Sample preparation and spectrometry analysis

Plasma samples were prepared by protein precipitation method and analyzed as we previously described^25^. Briefly, fifty µL of sample was transferred to an Eppendorf tube and 200 µL of cold methanol was added. After a slow agitation for one minute, samples were incubated for 30 min at -20 °C. Samples were then vortex mixed for one minute, centrifuged for 15 min at 4 °C, 11,000 rpm, and the supernatants were transferred to 10 kDa centrifugal filters (516 − 0230, VWR, Fontenay-sous-Bois, France) and centrifuged again for 45 min (conditions). Filtered extract was dried under the gentle nitrogen flux ant the dry extracts were dissolved into 125 µL of 10% acetonitrile / 0.1% formic acid solution. Extracts were finally filtered through 0.45 μm centrifugal filters for 15 min at 11,000 rpm, 4 °C and then 50 µL were transferred into new vials with inserts. Quality control samples (‘pooled samples’) were prepared by combining aliquots from each plasma sample and were interspersed at regular intervals, every 5 samples, throughout the analytical series. Blank samples were prepared using the same protocol as of the plasma samples and deionized water instead of plasma sample.

Plasma samples were analyzed separately using reverse phase and hydrophilic interaction liquid chromatography hyphenated to high-resolution mass spectrometry (LC-MS). Chromatographic separation was carried out on a Dionex UltiMate 3000 (Thermo Fisher Scientific, Bremen, Germany) operated by Chromeleon 6.8 software. Five µL of plasma sample were injected on each column and samples were maintained at 4 °C. Reverse phase column used was Hypersil Gold (100 mm x 2.1 mm x 1.9 μm) (Thermo Scientific). Column was kept at 40 °C and the flow rate was maintained at 400 µL/mL. Mobile phases were 0.1% formic acid solutions in water (A) and acetonitrile (B). For the first minute a 0% of B was kept isocratic followed by ten minutes of linear gradient up to 100% B which was maintained in isocratic mode for two minutes. Initial conditions were reached in one minute and the column equilibrated for two minutes. For the hydrophilic interaction separation, a Se-Quant, ZIC-HILIC Peek Coated (150 × 2.1 mm x 5 μm), column (Merck, Darmstadt, Germany) was used, kept at 25 °C with a flow rate at 250 µL/min. Mobile phases were 16 mM ammonium formate solution in water (A) and 0,1% formic acid solution in acetonitrile (B). For the first two minutes a 97% of B was kept isocratic followed by 8 min gradient to the 70% of B and 5 min gradient to the 10% of B. Return to the initial conditions was made in one minute with a 400 µL/min flow rate and maintained at that rate for 3 min. After that the initial flow rate, 250 µL/min was maintained for one minute.

Mass spectrometry analysis was performed on the Q-Exactive Plus hybrid mass spectrometer (Thermo Fisher Scientific) with a Heated Electrospray Ionization (H-ESI II) probe working in the switching polarities modes. Spray voltage was maintained at 3500 V. Transfer capillary was heated to 320 °C, sheath ad auxiliary gas flow rates were maintained at 30 and 8 arbitrary units and the gas was heated to 310 °C. S-lens RF was kept at 55 V to transfer ions of large mass-to-charge ratio (*m/z*) range toward the orbitrap mass analyser. Mass spectra were acquired in the 80-1000 *m/z* range for the reverse phase and 67–800 *m/z* for the hydrophilic interaction phase. Each scan was acquired by collecting the ions into the C-trap during up to 250 ms in order to obtain spectra with 35,000 FWHM (Full Width Half Maximum) resolving power for the theoretical *m/z* 200. Instrument setup and LC-MS system control were achieved under Thermo Xcalibur 3.0.63 software and the control of the mass spectrometer was achieved using Tune Q Exactive Plus 2.5 application.

Analytical batches were run as follows. First, blank samples were analysed in five replicates. Then 10 pooled samples were injected for the equilibration step, followed by experimental samples ran in random and interspaced every five samples by one pool sample. At the end a pool sample was submitted to High Collision Dissociation (HCD) experiment using Data Dependent Analysis in order to obtain structural information from the MS/MS spectra.

### Data processing

Acquired spectra were converted into mzXML files using ProteoWizard application which separates positive and negative ionisation mode files. Data were extracted using XCMS library under the R environment (Rx64 3.6.0). Peak detection was performed using the centWave method. For plasma samples XCMS parameters were as follows: chromatographic peak width was set from 3 to 30 s for reverse phase and 4–80 s for hydrophilic interaction phase, signal to noise threshold at 3, while the noise was set to 60,000 for reverse phase for both positive and negative ionisation modes, and 20,000 and 10,000 for hydrophilic interaction phase in positive and negative ionisation modes, respectively. For both types of columns, the *m/z* tolerance between two consecutive scans was set to 5 ppm. Prefilter of detected peaks was set to 4 consecutive scans with intensities three time higher the signal-to-noise level. Peak alignment between samples and peak grouping were achieved using the Obiwarp algorithm and density method^26^, respectively.

Extracted data matrices were then filtered in order to eliminate the analytical background and correct the analytical drift. Peaks coming from the blank samples and from the instrumental noise were removed from the matrix as well as the peaks that variated for more than 30% in all the pool samples (see Supplementary Fig. 1). For peak annotation, two data tables generated from the reversed-phase and HILIC analyses, containing full MS and retention time information, were compared to a local database that holds the same kind of data for 1,300 metabolites. Features annotated within 5 ppm error in mass precision and 0.5 min for the retention time drift were taken into farther examination. The adducts of the annotated metabolites were further correlated using a regression threshold of 0.8 to reduce the risk of false annotations. Redundant metabolites detected in both positive and negative ionization modes, as well as in both C18 and HILIC methods, were filtered based on the lowest coefficient of variation in QC samples. Consistent features were then checked concerning MS/MS spectra. Possible annotations were confirmed comparing the experimental MS/MS spectra to those of the reference standards of proposed molecules. Following all the workflow, an annotation/identification level was attributed to each feature, going from I to IV with slight modifications^27^. A level I is attributed to the unambiguous identification of molecule, based on the robust match of *m/z*, retention time and fragmentation pattern. A level II is attributed to the features with the high similarities in *m/z*, retention time and fragmentation pattern but with unambiguity of isomers or with a good match in the full scan spectra and retention time, but with a lack of fragmentation pattern. Level III represents a good match of *m/z* and retention time, and a presence of several adduct ions in the full scan spectra, but multiple concurrent propositions for the annotation and level IV concerns the annotations based only on *m/z* and retention times similarities (see supplementary Table 1).

Matching was performed using Workflow4Metabolomics^28^, using the LC-MS annotation with bank inhouse tab. Final plasma matrix contained 232 metabolites identified with different levels of identification for the broad-spectrum profiling, and 180 metabolites for the SSA study^29^.

### Enrichment analysis

Metabolomic data were analyzed and discriminant variables were interpreted using Metaboanalyst 6.0^30^. Small Molecule Pathway Database (https://smpdb.ca) was used with the specific MetaboAnalyst module (https//www.metaboanalyst.ca) to analyze pathway enrichment and discriminate data according to their involvement in metabolic and biological functions^31,32^.

### Statistical analysis

We conducted a sparse multiblock partial least square discriminant analysis (DIABLO sPLS-DA)^33^ to integrate the double scale blocks available in our data: a molecular block with metabolite levels and a macro block with CRP, injury score and skin perfusion with respect to the radiation exposure group. More precisely, DIABLO consists of a regularized and sparse generalized canonical correlation analysis which maximize the joint correlation between the indicator matrix group of radiation dose and the two blocks described above. Thus, it permits to achieve a double goal by performing simultaneously dose group classification and inter-block correlation tasks.

In this approach, the inter-block associations are captured through few new covariates called loadings constructed as a linear combination from the original variables of each block. In order to obtain an interpretable multi-scale signature, the loss function of this dimension reduction model incorporates a lasso penalization to select the most relevant variables of each block (in particular the metabolites) in order to avoid overfitting and ensure model stability. The hyperparameter of the model (number of loadings and their constituting variables) were defined using cross-validation selection (leave-one-out). Cross-validation is a model validation technique used in statistics and machine learning to assess the generalization performance of a learning method on independent test data. It consists in dividing the training data into several equal parts and learning the model on all parts except one for a range of values of the hyperparameters. In particular, the leave-one-out scheme restricts each part to a single observation. The performance (here classification and inter-block correlations) of the model is then evaluated on the remaining part of the data and the process is repeated in order to compute the averaged performance criterion over all the parts. Finally, the optimal hyperparameters are those associated with the model with the optimal performance^34^. All DIABLO analyses were performed using the mixOmics package (version 6.18.1)^35^ in R software.

## Results

### Physiopathology

**Fig. 1:**
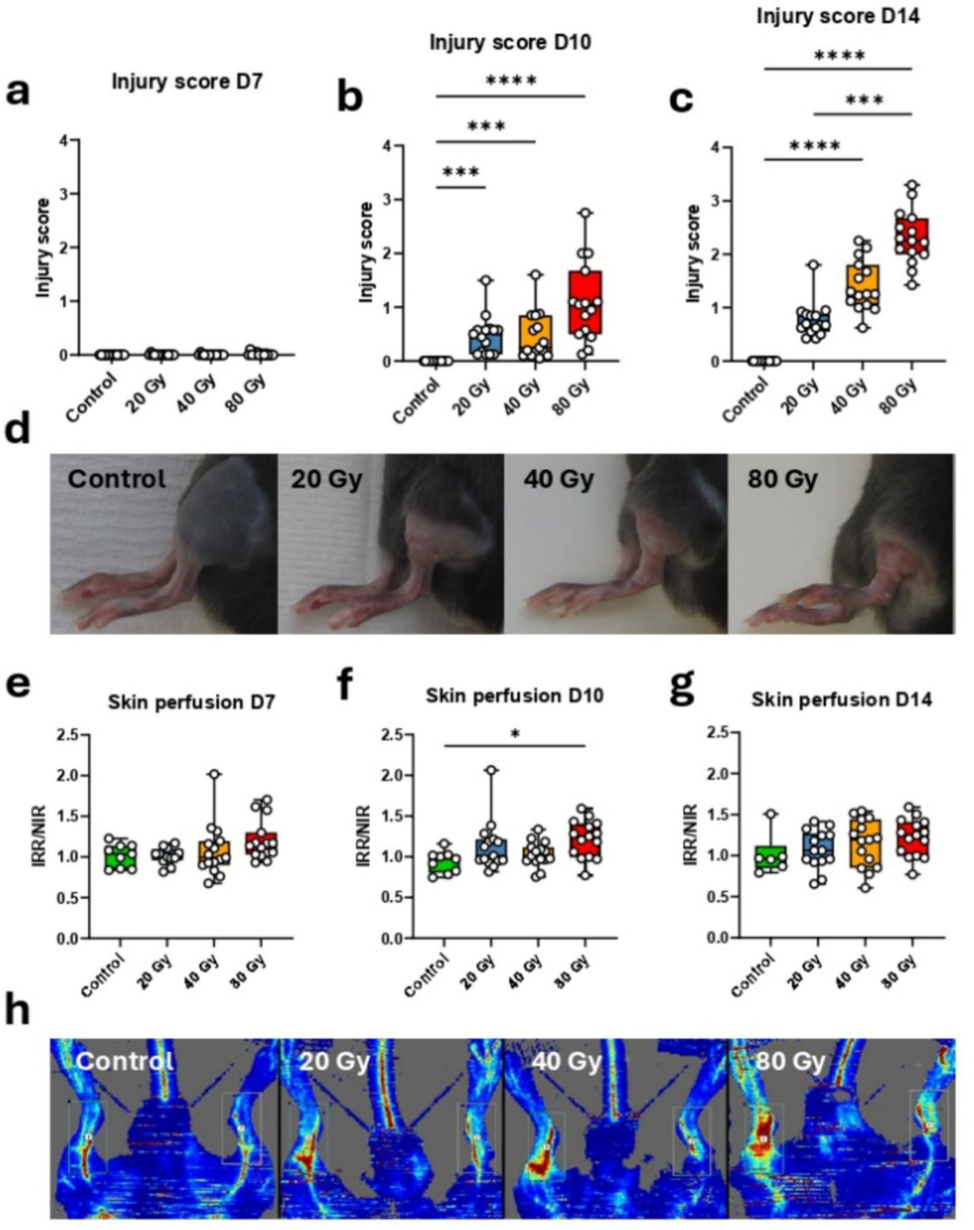
Physiopathology. Mean of observational injury score for each dose group assessed at day 7 (**a**) 10 (**b**) and 14 (**c**) post-irradiation. Representative illustration of the aspect of the injury (irradiated limb) at day 14 post-irradiation for each dose group (**d)**. Ratio of the mean skin perfusion of irradiated and non-irradiated limbs (IRR/NIR) for each dose group at day 7 (**e**), 10 (**f**) and 14 (**g**) post-irradiation. (**h**) Scans acquired with Doppler imagery for the assessment of skin perfusion at day 14 post-irradiation for all dose groups. Each animal is represented with a white point. Control *N* = 6–15, 20 Gy, 40 Gy, 80 Gy *N* = 15. Kruskal-Wallis test * *p-value* < 0.05, *** *p-value* < 0.001, **** *p-value* < 0.0001.

In our LRI preclinical model, there was no sign of injury in any dose group at day 7 after irradiation (Fig. 1a). At day 10, the injury appeared for all dose groups compared to the control group (*p-value* ≤ 0.0005) but showed no difference of severity between irradiated groups (Fig. 1b). At day 14 post-irradiation (end of experiment), the observational injury score showed a trend of increasing severity with increasing dose (Fig. 1c, d). As previously observed on the same preclinical model^12^, the average injury score of the 40 Gy group was not statistically different from the 20 and the 80 Gy groups despite a fold change of 1.85 and 0.61, respectively. Indeed, mice from the 40 Gy group displayed intermediate injury severity with individual heterogeneity: mice could show a severity profile close to the 20 Gy group, or to the 80 Gy group, or in-between. The laser Doppler analysis showed no difference for skin perfusion of irradiated limbs for any dose group 7 days after the irradiation (Fig. 1e). At day 10 post-irradiation, the skin perfusion was significantly higher in the irradiated limb of mice from the 80 Gy group compared to the control group (*p-value* < 0.05) (Fig. 1f). However, at day 14 post-irradiation, despite a slight trend of increasing perfusion in irradiated limb, there was no significant difference between experimental groups (Fig. 1g, h).

### Broad-spectrum profiling

**Fig. 2:**
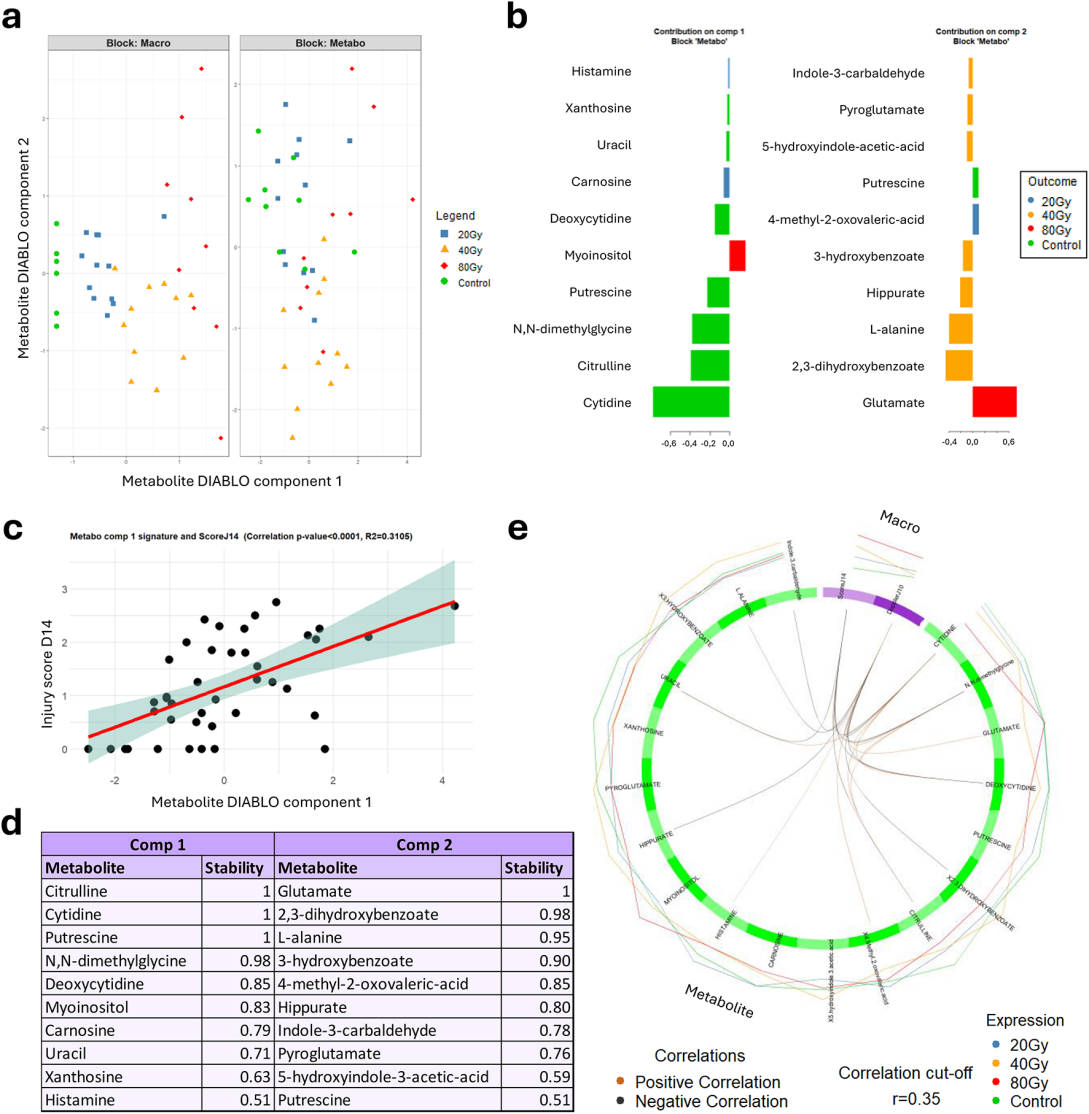
Broad-spectrum screening. (**a**) Mutiblock sPLS-DA scatter plots with “macro” block = injury score and skin perfusion and “metabo” block = plasma levels of 19 metabolites. (**b**) Graphs with the 19 metabolites from the two components with their loading values, representative of the contribution (coefficients in linear combination) of each metabolite for the segregation of one particular group (illustrated with the color). (**c**) Positive and significant correlation between the component 1 of the DIABLO model and the injury score at day 14 post-irradiation (p-value < 0.0001 and R² = 0.3105). (**d**) List of each metabolite of the panel (component 1 or 2) with their stability score. (**e**) Multi-scale correlations (estimated greater than 0.35) between the covariates of each block illustrated by orange (positive) or black (negative) line inside the circle plot. The “macro” block is represented in purple and the “metabo” block in green. Expression of the covariates according to the dose group is shown with colored line outside the circle. Control *N* = 9, 20 Gy *N* = 12, 40 Gy *N* = 11, 80 Gy *N* = 9.

A first cohort of mice was used for a broad-spectrum screening of metabolites in blood using LC-MS, in order to define a panel of metabolites with plasma levels enabling the separation of animals according to their dose group at day 14 post-irradiation. MS analysis led to the identification of 232 metabolites which were further analyzed using multivariate analysis. The sPLS-DA analysis revealed that the “macro” block (constructed with data from injury score and skin perfusion) allowed a clear segregation of mice according to their dose group (Fig. 2a). On the other hand, thanks to the Lasso penalization of the sPLS-DA (see methods), 19 metabolites were selected to jointly segregate dose groups and correlate to injury severity at day 14 post-irradiation. This “metabo” block, was able to separate the control group, the 40 Gy group, and the 80 Gy group (Fig. 2a). However, the 20 Gy group (Fig. 2a, blue squares) seems to be distributed across the control group and the 40 Gy group. Each metabolite of this panel had a preferential contribution for the segregation of one particular group from the others (Fig. 2b), with for example a negative weight for cytidine in component 1 with maximal median value in the control group, or a positive weight of glutamate in component 2 with maximal median value in the 80 Gy group. The component 1 of the plasma metabolomic panel was significantly correlated with the injury score of the LRI at day 14 post-irradiation (*p-value* < 0.0001) (Fig. 2c).

The complete panel of metabolites from components 1 and 2 associated with their stability score is listed on Fig. 2d. This stability score informs how often the same metabolite is selected when the training set is perturbed via leave-one-out cross-validation. This panel corresponds to our preliminary signature of plasma metabolites jointly associated with radiation exposure level and injury severity. To go further, the multi-scale correlations between plasma metabolite levels, injury severity and skin perfusion modelled by the DIABLO model are illustrated on Fig. 2e. Interestingly, uracil, deoxycytidine and citrulline are shown to be correlated with injury severity at day 14 post-irradiation, when other metabolites including hippurate, L-alanine and glutamate correlate with cutaneous blood perfusion at day 10 post-irradiation. Furthermore, metabolites display correlations between each other’s, especially cytidine and dimethyglycine which show multiple connections with other metabolites. Finally, an enrichment analysis based on these 19 identified metabolites revealed glutathione metabolism, urea cycle, beta-alanine metabolism, glucose-alanine cycle and histidine, alanine and pyrimidine metabolism as most enriched pathways (Supplementary Fig. 2).

### Suspect screening analysis (SSA)

In order to refine this first-stage signature for the diagnosis of LRI in mice plasma, a second independent cohort of mice was used to perform a SSA study based on the previously identified signature. More precisely, an untargeted metabolomic analysis was conducted on the independent mice cohort, then the obtained data matrix was restricted to the 19-metabolite panel highlighted in the broad-spectrum study.

**Fig. 3:**
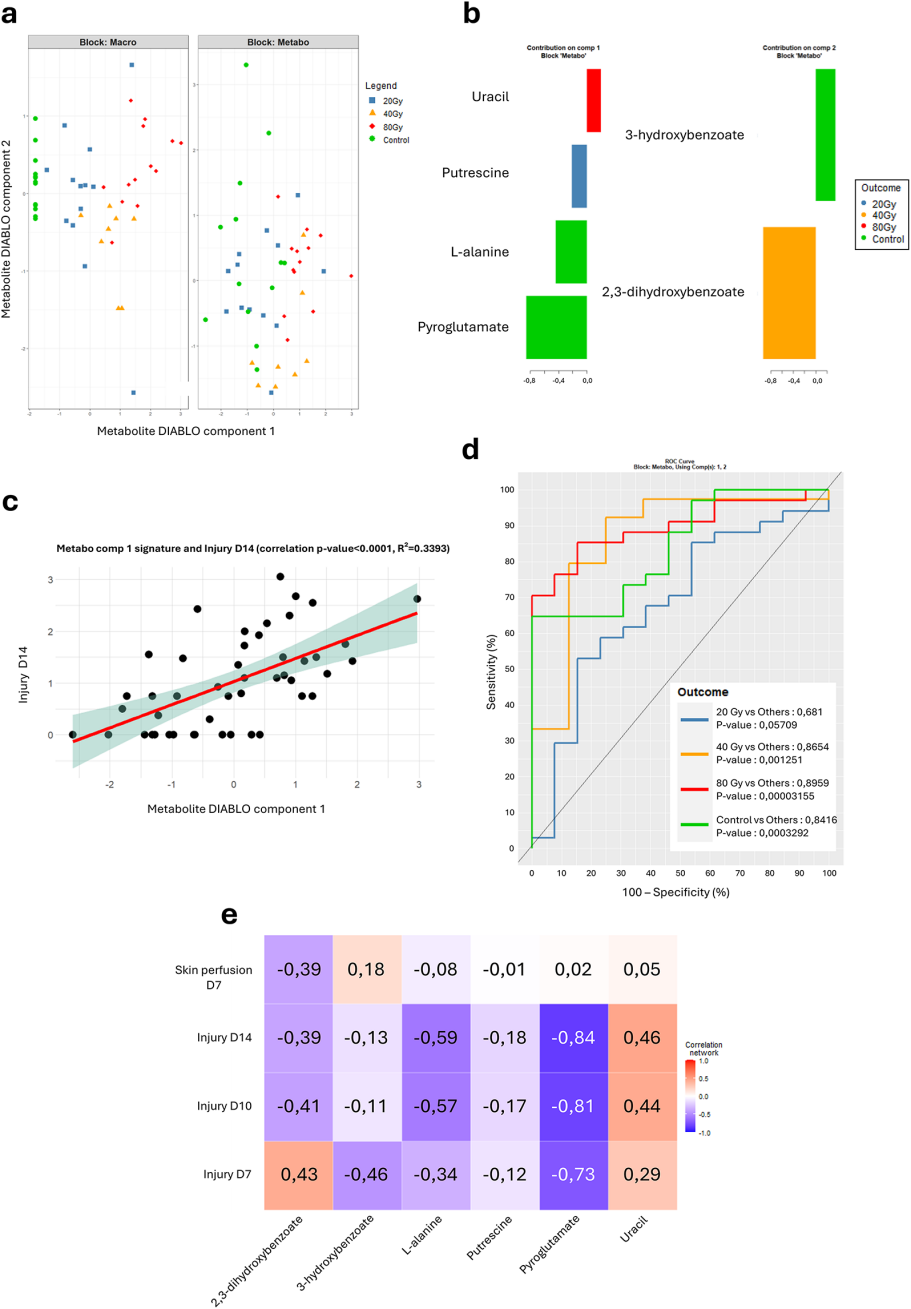
Suspect screening analysis. (**a**) Mutiblock sPLS-DA scatter plots with “macro” block = injury score and skin perfusion and “metabo” block = plasma levels of 6 metabolites. (**b**) Loading plots of the 6 metabolites with their contribution in the component. (**c**) Positive and significant correlation between the component 1 of the DIABLO model and the injury score at day 14 post-irradiation (p-value < 0.0001 and R² = 0.3393). (**d**) ROC curves illustrating the performance of the signature to separate each group from the others (AUC) with the associated p-value. (**e**) Heat-map of the correlations between plasma levels of the metabolites from the signature and injury severity at day 7, 10 and 14 and skin perfusion at day 7 post-irradiation, with positive correlations in red and negative correlations in blue. Control *N* = 6, 20 Gy *N* = 11, 40 Gy *N* = 11, 80 Gy *N* = 9.

As explained above, the DIABLO modelling performed a new variable selection process to avoid overfitting and reduce from 19 to 6 metabolites in the SSA study signature. The two-dimensional projection of the DIABLO multi-block scores showed the ability of this 6-metabolite subpanel to separate the control group, the 40 Gy group and the 80 Gy group at day 14 post-irradiation (Fig. 3a). However, mice from the 20 Gy group were distributed among the other groups and did not show a clear segregation. The preferential contributions of each of these 6 metabolites to separate one group from the others are shown on Fig. 3b. The first component of metabolites signature displayed significant positive correlation with injury severity at day 14 (R²=0.34, *p-value* < 0.0001) (Fig. 3c). Receiving operator characteristics (ROC) curves showed great performances of the 6-metabolite signature to separate the control group from all the others, with an area under the curve (AUC) of 0.84 (p-value = 0.00033), as well as the 40 Gy group (AUC = 0.86, p-value = 0.00125) and the 80 Gy group, with an AUC = 0.89 (p-value < 0.0001, Fig. 3d). However, the signature achieved lower ability to separate the 20 Gy group from the others with border significance AUC = 0.68 (p-value = 0.05709). The heat map shows positive and negative correlations between plasma levels of the 6 metabolites and injury parameters such as injury severity at day 7, 10 and 14 post-irradiation and skin perfusion at day 7 post-irradiation (Fig. 3e). Notably, L-alanine, pyroglutamate (5-oxoproline) and uracil show interesting correlations with injury severity from day 10 to day 14 post-irradiation, and even since day 7 for pyroglutamate (injury scores at day 7 correspond to erythema in some mice from the 40 and 80 Gy groups in this cohort). The fold changes of the 6 metabolites comprising this diagnostic signature, in relation to the dose, are presented in Supplementary Fig. 3.

Altogether, the 6 metabolites identified in this study constitute a final mouse plasma signature of the LRI in our preclinical model (Table 1). This 6-metabolite signature can efficiently distinguish irradiated from non-irradiated mice and allows the segregation of animals according to their radio-induced injury severity.

**Table 1:** Diagnostic signature. List of the 6 metabolites that compose the diagnostic signature of the LRI in mice plasma with their analytical parameters and their putative functions, according to literature.

Metabolite	LC-MS mode	Detected ions	Precursor m/z (error, ppm)	Product m/z	Retention time, min (delta RT)	Putative function	References
Putrescine (HMDB0001414)	HILIC, positive H-ESI	[M + H]^+^,	89.1073 (0.5)	72.0807	17.05 (0.35)	Related to hair loss and skin histology Anti-inflammatory and antioxidant effects in rats with brain ischemia-reperfusion	Peralta et al., 1996 Dasdelen, 2023^55^
		[M + H-NH3]^+^	72.0808 (0.5)		17.05 (0.35)		
Uracil (HMDB0000300)	HILIC, negative H-ESI	[M-H]^−^	111.0200 (0.36)	41.9985	2.32 (0.09)	DNA damage Increased with dose in human skin irradiation	Chen et al., 2024^52^ Hu et al., 2012^47^
2,3-Dihydroxybenzoate (HMDB0000397)	HILIC, negative H-ESI	[M-H-CO2]^−^	109.0293 (2.7)		2.21 (0.07)	Inflammatory and antioxidant activities	Chen et al., 2022
		[M-H]^−^	153.0194 (0.3)	109.0295	2.20 (0.06)		
3-Hydroxybenzoate (HMDB0002466)	HILIC, negative H-ESI	[M-H]^−^	137.0244 (0.2)	93.0346	1.92 (0.23)	Anti-oxidant activity	Zhao et al., 2018^57^
l-Alanine (HMDB0000161)	HILIC, positive H-ESI	[M + H]^+^	90.0550 (0.8)	44.0495	13.77 (0.03)	Related to inflammation in patients with rhumatoid arthritis	Narasimhan et al., 2018^58^
Pyroglutamate (5-oxoproline) (HMDB0000267)	C18, positive H-ESI	[M + H]^+^	130.0500 (0.6)	84.0451	0.81 (0.00)	Pro-inflammatory activity, increasing DNA damages Deregulated in irradiated porcine skin Deregulated in muscle cells after irradiation	Li et al., 2022^54^ Burnett et al., 2019^23^ Wang et al., 2016^61^

## Discussion

With the objective to identify early metabolite prognostic biomarkers of LRI in plasma, i.e., before the appearance of clinical symptoms, we first aimed to provide the proof of concept of the use of plasma metabolites as LRI biomarkers by identifying a metabolomic diagnostic signature associated with established injury in a preclinical model. Moreover, such signature would help with LRI diagnosis by providing additional information relating to the severity of the injury, since molecular modifications are frequently more sensitive and predictive than phenotypic macroscopic observations^36^.

IR alters the good functioning of cells and tissues by inducing inflammation, oxidative stress, mitochondrial dysfunction, and DNA damages leading to variations of metabolites concentrations, which makes them good candidates as irradiation biomarkers^13^. On this basis, several studies have already shown the interest of metabolites in urine, serum, and saliva as biomarkers of IR exposure in large body area irradiation models (TBI and Partial Body Irradiation, PBI) using mice, rats and NHPs^13–15^. Notably, Pannkuk & al documented several deregulated metabolites in NHPs after 2–10 Gy TBI in urine and serum up to 7 days after exposure^21,37^. In mice, plasma citrulline^38^ was established as a biomarker of gastrointestinal acute radiation syndrome (GI-ARS) after TBI, while other metabolite signatures have been identified in urine following X-rays exposure (TBI)^39^ or for internal contamination with cesium-137^16^. Interestingly, some metabolites already identified in animals displayed urinary deregulation in patients after a TBI administered before a bone marrow transplant^40^. Furthermore, metabolites changes in irradiated skin tissue have also been demonstrated in a porcine model of LRI^23^.

In the present study, we used the same preclinical model as previously described^12^ to explore the potential of plasma metabolites at reflecting LRI development and severity. Animals showed an increasing injury severity with increasing dose, so that the average injury scores were significantly different between control, 20 Gy and 80 Gy groups, although the 40 Gy group could not be significantly distinguished from other irradiated groups at the presented timepoints, as previously observed^12^. Additionally, the assessment of skin perfusion was only able to detect the highest group of irradiation at day 10 post-irradiation, and therefore may be used only as a complementary tool for the diagnostic of extremely high-injured individuals. We also previously demonstrated that in this model, usual biological analysis classically performed for the detection of acute TBI, such as complete blood counts and CRP, do not display any difference among dose groups^12^. These results point out the necessity to identify more sensitive biomarkers to help with LRI diagnosis. Since metabolites are a real-time reflection of the current phenotype and functional response after an external stress such as irradiation, they appear to be excellent candidate biomarkers of various pathologies, including LRI^13–15^.

To explore this hypothesis, we used a two-step experimental strategy including two independent cohorts of mice. The first cohort served for the broad-spectrum profiling resulting in the identification of 232 metabolites in plasma, which led to the selection of a panel of 19 metabolites allowing a correct segregation of all groups of mice. Moreover, the 19-metabolite signature presents a significant correlation with the injury severity at day 14 postirradiation, which provides the proof of concept of the use of plasma metabolites as systemic biomarkers of the LRI. Interestingly, modulation of some of these metabolites, including xanthosine^41^, deoxycytidine^42^, and hippuric acid^43^, has already been shown in mice urine after TBI, while citrulline has been identified as serum biomarker of GI-ARS following PBI in mice^44–46^. Other metabolites were found deregulated in human skin tissue following irradiation (myo-inositol^47^) or in inflammatory skin disorders (histamine^48^). Although preliminary, enrichment analysis using this 19-metabolite list revealed that most modulated pathways in our model included glutathione metabolism, urea cycle, beta-alanine and pyrimidine metabolism. Interestingly, those pathways are related to pathological conditions involving inflammation^49^, oxidative stress^50,51^, and DNA damage^52^, which are part of LRI physiopathology and are known to be deregulated following irradiation^13,14^.

As the objective of this study was the identification of a robust metabolite biomarker signature of LRI, we then aimed to refine the broad-spectrum signature through a SSA study in order to validate its relevance for LRI diagnosis with a second investigation in an independent animal cohort.

Results from the SSA study unveiled a 6-metabolite signature correlated with injury severity at day 14 and able to distinguish animals from control, 40 and 80 Gy groups. The performances of the 6-metabolite panel to discriminate the experimental groups from one another were highly significant to ensure the relevance of the signature for the diagnosis of LRI. As with the preliminary signature from the broad-spectrum analysis, in our model, animals from the 20 Gy group were less distinguishable from the others although the AUC was close to significance (*p-value* = 0.06). These observations suggest that this 6-metabolite signature is more efficient at discerning animals with moderate and severe injuries, while it is more challenging to identify mice experiencing slighter injuries, even from controls. Interestingly, the plasma miRNA signature of LRI described in our earlier study did not provide strong performance at discriminating mice from the 40 Gy group from the others but successfully separated controls from slight injury group exposed at 20 Gy^12^. These results suggest the potential complementarity of metabolite and miRNA approaches to distinguish animals according to injury severities. The assumption of using both circulating miRNA and metabolites to improve LRI diagnosis is even more interesting considering that the combination of those two omics biomarkers is not so usual in radiobiology^53^.

This study described a 6-metabolite plasma signature associated with LRI severity which could provide a picture of multi-scale molecular and cellular modifications commonly induced by irradiation in mice. Indeed, while uracil^52^ and pyroglutamate^54^ were related to DNA damage response, putrescine^55^, 2,3-dihydroxybenzoate^56^ and 3-hydroxybenzoate^57^ have been described with anti-oxidant and/or anti-inflammatory activities. Furthermore, serum L-alanine^58^ has been associated with inflammation markers in patients with rheumatoid arthritis; putrescine^59^ with hair loss and damaged skin structure; and pyroglutamate^60^ was identified as potential biomarker of myocardial injury. In addition, putrescine was highlighted as a potential urinary biomarker of TBI sublethal radiation exposure in mice^41^, while uracil^47^ and pyroglutamate^61^ were shown to be deregulated in human skin tissue and muscle cells, respectively, after irradiation. Interestingly, in a pig model of cutaneous radiation injury^23^, a decrease in pyroglutamate (5-oxoproline) production in skin biopsies exposed to the highest dose (37 Gy) was observed up to 70 days post-irradiation. Furthermore, 5-oxoproline is also related to glutathione metabolism, e.g. oxidative stress defense potential^62^.

Despite the presence of a fully developed injury, which limits the interest of the signature beyond a complementary diagnostic tool, this study supports the use of plasma metabolites as LRI biomarkers and paves the way for a prognostic signature of the injury. The suggested complementarity of metabolite and miRNA signatures for the diagnosis of LRI provides strong arguments for an integrated study using both candidate biomarkers to identify a composite signature with potentially higher efficiency. In the same way, a validation of the signature has to be done in other species such as rats or minipigs. Before considering exploring metabolites signature in humans, and in regard to metabolites variations between male and female^37,40^, a study should be performed on both sexes in order to identify a universal signature or, in contrary, sex-specific signatures.

In conclusion, we provided the preclinical proof of concept for the use of metabolites as plasma biomarkers for molecular diagnosis of LRI and identified a metabolite signature of the injury in mice plasma. Each metabolite from this panel is related to molecular or cellular dysfunctions known to be induced by radiation exposure. This metabolite signature could constitute a potential complementary diagnostic tool for the assessment of LRI severity, in industrial or medical accident cases. Relying on those promising results, a metabolomic prognostic study is ongoing in our laboratory in order to determine early plasma signature of LRI before the appearance of the first symptoms.

## Electronic supplementary material

Below is the link to the electronic supplementary material.

Supplementary Material 1

Supplementary Material 2

Supplementary Material 3

Supplementary Material 4

Supplementary Material 5

## Data Availability

All relevant data are included in the paper and/or its supplementary information files.

## References

[1] International Atomic Energy Agency. *Generic Procedures for Medical Response during a Nuclear or Radiologic Al Emergency* (Emergency Preparedness and Response, 2005).

[2] Bray, F. N., Simmons, B. J., Wolfson, A. H. & Nouri, K. Acute and chronic cutaneous reactions to Ionizing Radiation Therapy. *Dermatol. Ther.* **6**, 185–206 (2016). 10.1007/s13555-016-0120-y PMC4906114 27250839

[3] Lataillade, J. New approach to radiation burn treatment by dosimetry-guided surgery c ombined with autologous mesenchymal stem cell therapy. *Regen Med.* **2**, 785–794 (2007). 17907931 10.2217/17460751.2.5.785

[4] Ryan, J. L., Ionizing & Radiation The Good, the bad, and the Ugly. *J. Invest. Dermatol.* **132**, 985–993 (2012). 22217743 10.1038/jid.2011.411 PMC3779131

[5] 5 International Atomic Energy Agency. Medical management of radiation injuries. *Saf. Rep. Ser.* (2020).

[6] Huchet, A. Plasma Flt-3 ligand concentration correlated with radiation-induced bo ne marrow damage during local fractionated radiotherapy. *Int. J. Radiat. Oncol. Biol. Phys.* **57**, 508–515 (2003). 12957264 10.1016/s0360-3016(03)00584-4

[7] Shim, S. Mitigating effects of hUCB-MSCs on the hematopoietic syndrome resultin g from total body irradiation. *Exp. Hematol.* **41** (342), 346–353 (2013). 23333483 10.1016/j.exphem.2013.01.002

[8] Bujold, K. Citrulline as a biomarker for gastrointestinal-Acute Radiation Syndrom e: species differences and experimental Condition effects. *Radiat. Res.* **186**, 71–78 (2016). 27351760 10.1667/RR14305.1 PMC4976929

[9] Kumar, P. Evaluation of plasma biomarker utility for the gastrointestinal acute radiation syndrome in non-human primate after partial body irradiation with minimal bone marrow sparing through correlation with tissue and histological analyses. *Health Phys.* **119**, 594–603 (2020). 32947487 10.1097/HP.0000000000001348 PMC7546578

[10] Chaze, T. Serum Proteome Analysis for Profiling Predictive Protein Markers Assoc iated with the severity of skin Lesions Induced by Ionizing Radiation. *Proteomes* **1**, 40–69 (2013). 28250398 10.3390/proteomes1020040 PMC5302747

[11] Guipaud, O. Time-course analysis of mouse serum proteome changes following exposur e of the skin to ionizing radiation. *PROTEOMICS* **7**, 3992–4002 (2007). 17960731 10.1002/pmic.200601032

[12] Ancel, L. microRNA blood signature for localized radiation injury. *Sci. Rep.* **14**, 2681 (2024). 38302506 10.1038/s41598-024-52258-2 PMC10834964

[13] Hladik, D., Bucher, M., Endesfelder, D. & Oestreicher, U. The potential of Omics in Biological Dosimetry. *Radiation* **2**, 78–90 (2022).

[14] Shakyawar, S. K. A review of Radiation-Induced alterations of multi-omic profiles, Radi Ation Injury biomarkers, and countermeasures. *Radiat. Res.* **199**, 89–111 (2022). 10.1667/RADE-21-00187.1 PMC10279411 36368026

[15] Vicente, E., Vujaskovic, Z. & Jackson, I. L. A systematic review of metabolomic and lipidomic candidates for Biomar kers in Radiation Injury. *Metabolites* **10**, 259 (2020). 32575772 10.3390/metabo10060259 PMC7344731

[16] Goudarzi, M. Development of urinary biomarkers for internal exposure by Cesium-137 using a Metabolomics Approach in mice. *Radiat. Res.* **181**, 54–64 (2013). 24377719 10.1667/RR13479.1 PMC4029349

[17] Grison, S. The Metabolomic Approach identifies a Biological signature of low-dose chronic exposure to Cesium 137. *J. Radiat. Res. (Tokyo)*. **53**, 33–43 (2012). 22302043 10.1269/jrr.11071

[18] Grison, S. Metabolomics reveals dose effects of low-dose chronic exposure to Uran Ium in rats: identification of candidate biomarkers in urine samples. *Metabolomics* **12**, 154 (2016). 27729830 10.1007/s11306-016-1092-8 PMC5025510

[19] Menon, S. S. Radiation Metabolomics: current status and future directions. *Front. Oncol.* **6**, 20 (2016). 26870697 10.3389/fonc.2016.00020 PMC4736121

[20] Maan, K., Tyagi, R., Dutta, A., Bakhshi, R. & Rana, P. Comparative metabolic profiles of total and partial body radiation exp osure in mice using an untargeted metabolomics approach. *Metabolomics* **16**, 124 (2020). 33245511 10.1007/s11306-020-01742-7

[21] Pannkuk, E. L. Liquid Chromatography–Mass Spectrometry-based metabolomics of Nonhuman Primates after 4 gy total body Radiation exposure: global effects and targeted panels. *J. Proteome Res.* **18**, 2260–2269 (2019). 30843397 10.1021/acs.jproteome.9b00101 PMC6559376

[22] Zhao, H. Identification of potential Radiation Responsive metabolic biomarkers in plasma of rats exposed to different doses of Cobalt-60 Gamma rays. *Dose-Response* **18**, 1559325820979570 (2020). 33402881 10.1177/1559325820979570 PMC7745571

[23] Burnett, L. R. Biomolecular Analysis of Beta Dose-Dependent Cutaneous Radiation Injur y in a Porcine Model. *Radiat. Res.* **192**, 145–158 (2019). 31166846 10.1667/RR14283.1

[24] Tamarat, R., Lataillade, J. J., Bey, E., Gourmelon, P. & Benderitter, M. Stem cell therapy: from bench to bedside. *Radiat. Prot. Dosimetry*. **151**, 633–639 (2012). 22969031 10.1093/rpd/ncs160

[25] Chikh, K. Early metabolic disruption and predictive biomarkers of delayed-Cerebr Al Ischemia in Aneurysmal Subarachnoid Hemorrhage. *J. Proteome Res.* **23**, 316–328 (2024). 38148664 10.1021/acs.jproteome.3c00575

[26] Smith, C. A., Want, E. J., O’Maille, G., Abagyan, R. & Siuzdak, G. X. C. M. S. Processing Mass Spectrometry Data for Metabolite Profiling Usin g nonlinear peak alignment, matching, and identification. *Anal. Chem.* **78**, 779–787 (2006). 16448051 10.1021/ac051437y

[27] Lin, L. et al. LC-MS based serum metabonomic analysis for renal cell carcinoma diagnosis, staging, and biomarker discovery. *J. Proteome Res.* **10**, 1396–1405 (2011). 21186845 10.1021/pr101161u

[28] Giacomoni, F. Workflow4Metabolomics: a collaborative research infrastructure for com putational metabolomics. *Bioinformatics* **31**, 1493–1495 (2015). 25527831 10.1093/bioinformatics/btu813 PMC4410648

[29] Rochat, B. Proposed confidence scale and ID score in the identification of known- unknown compounds using high Resolution MS Data. *J. Am. Soc. Mass. Spectrom.* **28**, 709–723 (2017). 28116700 10.1007/s13361-016-1556-0

[30] Pang, Z. MetaboAnalyst 6.0: towards a unified platform for metabolomics data processing, analysis and interpretation. *Nucleic Acids Res.* **52**, 398–406 (2024). 10.1093/nar/gkae253 PMC11223798 38587201

[31] Frolkis, A. S. M. P. D. B. The small molecule pathway database. *Nucleic Acids Res.* **38**, 480–487 (2010). 10.1093/nar/gkp1002 PMC2808928 19948758

[32] Jewison, T. SMPDB 2.0: big improvements to the small molecule pathway database. *Nucleic Acids Res.* **42**, 478–484 (2014). 10.1093/nar/gkt1067 PMC3965088 24203708

[33] Singh, A. DIABLO: an integrative approach for identifying key molecular drivers from multi-omics assays. *Bioinforma Oxf. England)*. **35**, 3055–3062 (2019). 10.1093/bioinformatics/bty1054 PMC6735831 30657866

[34] Hastie, T., Tibshirani, R. & Friedman, J. H. *The Elements of Statistical Learning: Data Mining, Inference, and Prediction* (Springer, 2009).

[35] Rohart, F., Gautier, B., Singh, A. & Lê Cao, K. A. mixOmics: an R package for ‘omics feature selection and multiple data integration. *PLOS Comput. Biol.* **13**, e1005752 (2017). 29099853 10.1371/journal.pcbi.1005752 PMC5687754

[36] Chen, R. & Snyder, M. Promise of personalized omics to precision medicine. *Wiley Interdiscip Rev. Syst. Biol. Med.* **5**, 73–82 (2013). 23184638 10.1002/wsbm.1198 PMC4154620

[37] Pannkuk, E. L., Laiakis, E. C., Authier, S., Wong, K. & Fornace, A. J. Jr. Global metabolomic identification of long-term dose-dependent urinary biomarkers in Nonhuman Primates exposed to Ionizing Radiation. *Radiat. Res.* **184**, 121 (2015). 26230079 10.1667/rr14091.1 PMC4539133

[38] Jones, J. W. Citrulline as a Biomarker in the murine total-body irradiation model: correlation of circulating and tissue citrulline to small intestine ep ithelial histopathology. *Health Phys.* **109**, 452 (2015). 26425905 10.1097/HP.0000000000000346 PMC4727745

[39] Chen, C., Brenner, D. J. & Brown, T. R. Identification of urinary biomarkers from X-Irradiated mice using NMR spectroscopy. *Radiat. Res.* **175**, 622–630 (2011). 21338244 10.1667/RR2388.1

[40] Laiakis, E. C. Development of a Metabolomic Radiation Signature in urine from patient s undergoing total body irradiation. *Radiat. Res.* **181**, 350–361 (2014). 24673254 10.1667/RR13567.1 PMC4071158

[41] Manna, S. K., Krausz, K. W., Bonzo, J. A., Idle, J. R. & Gonzalez, F. J. Metabolomics reveals aging-associated attenuation of noninvasive radia tion biomarkers in mice: potential role of polyamine catabolism and in coherent DNA damage-repair. *J. Proteome Res.* **12**, 2269–2281 (2013). 23586774 10.1021/pr400161k PMC3678303

[42] Tyburski, J. B. Radiation Metabolomics. 2. Dose- and time-dependent urinary excretion of Deaminated purines and Pyrimidines after Sublethal Gamma-Radiation exposure in mice. *Radiat. Res.* **172**, 42–57 (2009). 19580506 10.1667/RR1703.1 PMC2794378

[43] Kurland, I. J. Integrative Metabolic Signatures for hepatic Radiation Injury. *PLOS ONE*. **10**, e0124795 (2015). 26046990 10.1371/journal.pone.0124795 PMC4457483

[44] Bertho, J. M. New Biological indicators to Evaluate and Monitor Radiation-Induced Da mage: an Accident Case Report. *Radiat. Res.* **169**, 543–550 (2008). 18439044 10.1667/RR1259.1

[45] Kumar, V. P. Development of a Multi-organ Radiation Injury Model with Precise Dosim etry with Focus on GI-ARS. *Radiat. Res.* **201**, 19–34 (2024). 38014611 10.1667/RADE-23-00068.1

[46] Lutgens, L. C. H. W. plasma citrulline concentration: a surrogate end point for radiation-i nduced mucosal atrophy of the small bowel. A feasibility study in 23 p atients. *Int. J. Radiat. Oncol.* **60**, 275–285 (2004). 10.1016/j.ijrobp.2004.02.052 15337566

[47] Hu, Z. P. Metabolomic response of human skin tissue to low dose ionizing radiati on. *Mol. Biosyst*. **8**, 1979–1986 (2012). 22610363 10.1039/c2mb25061f

[48] Kolkhir, P. & Urticaria *Nat. Rev. Dis. Primer* **8**, 1–22, (2022). 10.1038/s41572-022-00389-z 36109590

[49] Jelonek, K., Mrowiec, K., Gabryś, D. & Widłak, P. The metabolic footprint of systemic effects in the blood caused by rad iotherapy and inflammatory conditions: a. *Syst. Rev. Metab.* **13**, 1000 (2023). 10.3390/metabo13091000 PMC10534379 37755280

[50] Schnuck, J. K., Sunderland, K. L., Kuennen, M. R. & Vaughan, R. A. Characterization of the metabolic effect of β-alanine on markers of ox idative metabolism and mitochondrial biogenesis in skeletal muscle. *J. Exerc. Nutr. Biochem.* **20**, 34–41 (2016). 10.20463/jenb.2016.06.20.2.5 PMC4977905 27508152

[51] Shimura, T. Radiation affects glutathione redox reaction by reduced glutathione pe roxidase activity in human fibroblasts. *J. Radiat. Res. (Tokyo)*. **63**, 183–191 (2022). 34977941 10.1093/jrr/rrab122 PMC8944298

[52] Chen, J. & Exploring, D. N. A. Damage and Repair Mechanisms: A Review with Computationa l Insights. *BioTech* 13, 3, (2024). 10.3390/biotech13010003 PMC10801582 38247733

[53] Subedi, P., Moertl, S. & Azimzadeh, O. Omics in Radiation Biology: surprised but not disappointed. *Radiation* **2**, 124–129 (2022).

[54] Li, M. Discovery and Validation of Potential Serum Biomarkers with Pro-Inflam matory and DNA Damage Activities in Ulcerative Colitis: A Comprehensiv e Untargeted Metabolomic Study. *Metabolites* 12, 997, (2022). 10.3390/metabo12100997 PMC9609580 36295899

[55] Dasdelen, D., Cetin, N., Menevse, E., Baltaci, A. K. & Mogulkoc, R. Effects of putrescine on oxidative stress, spermidine/spermine-N(1)-ac etyltransferase, inflammation and energy levels in liver and serum in rats with brain ischemia-reperfusion. *Physiol. Int.* **110**, 34–45 (2023). 36800189 10.1556/2060.2022.00138

[56] Spiegel, M. Antioxidant activity of selected phenolic acids–Ferric reducing Antiox Idant Power Assay and QSAR Analysis of the structural features. *Molecules* **25**, 3088 (2020). 32645868 10.3390/molecules25133088 PMC7412039

[57] Zhao, Z., Vavrusova, M. & Skibsted, L. H. Antioxidant activity and calcium binding of isomeric hydroxybenzoates. *J. Food Drug Anal.* **26**, 591–598 (2018). 29567228 10.1016/j.jfda.2017.07.001 PMC9322242

[58] Narasimhan, R. Serum metabolomic profiling predicts synovial gene expression in rheum atoid arthritis. *Arthritis Res. Ther.* **20**, 164 (2018). 30075744 10.1186/s13075-018-1655-3 PMC6091066

[59] Soler, A. P., Gilliard, G., Megosh, L. C. & O’Brien, T. G. Modulation of murine hair follicle function by alterations in Ornithin E decarboxylase activity. *J. Invest. Dermatol.* **106**, 1108–1113 (1996). 8618048 10.1111/1523-1747.ep12340155

[60] Zhang, Y. Serum metabolism characteristics of patients with myocardial injury af ter noncardiac surgery explored by the untargeted metabolomics approac h. *BMC Cardiovasc. Disord*. **24**, 88 (2024). 38310264 10.1186/s12872-024-03736-y PMC10838454

[61] Wang, M., Keogh, A., Treves, S., Idle, J. R. & Beyoğlu, D. The metabolomic profile of gamma-irradiated human hepatoma and muscle cells reveals metabolic changes consistent with the Warburg effect. *PeerJ* **4**, e1624, (2016). 10.7717/peerj.1624 PMC4730869 26823999

[62] Yu, Y. M. & Plasma L-5-oxoproline kinetics and whole blood glutathione synthesis r ates in severely burned adult humans. *Am. J. Physiol. Endocrinol. Metab.* **282**, 247–258 (2002). 10.1152/ajpendo.00206.2001 11788355

