# Supplementary material for: Metabolomics identifies plasma biomarkers of localized radiation injury
Source: Sci Rep. 2025 Jan 16;15:2166. doi: 10.1038/s41598-025-85717-5 (PMC11739571; doi:10.1038/s41598-025-85717-5)
Supplement: Supplementary file 1 — Supplementary Material 1 [file 41598_2025_85717_MOESM1_ESM.docx]

**Supplementary figure 1:** PCA plots of plasma matrices from positive and negative ionization modes using HILIC and C18 columns after various data preprocessing steps: raw data, pool %RSD lower than 30% and S/N>5.

**Supplementary Figure 2** : Enrichment analysis

**Supplementary Figure 3**. Fold Changes of 6 metabolites from diagnostic signature. The fold changes of the 6 metabolites at day 14 post-irradiation in plasma are presented as means for each dose group. Each animal is represented with a white dot. Control N = 12, 20 Gy N = 13, 40 Gy N = 8, 80 Gy N = 12. Kruskal-Wallis test * *p-value* < 0.05, ** *p-value* < 0.01.

**Supplementary Table 1**: Metabolite annotations. Experimental group for each animal is indicated in the first column: CONT (control), 20 Gy, 40 Gy, or 80 Gy.
