# Supplementary figures and images for: Metabolomics identifies plasma biomarkers of localized radiation injury
Source: Sci Rep. 2025 Jan 16;15:2166. doi: 10.1038/s41598-025-85717-5 (PMC11739571; doi:10.1038/s41598-025-85717-5)

## Supplementary Figure 1

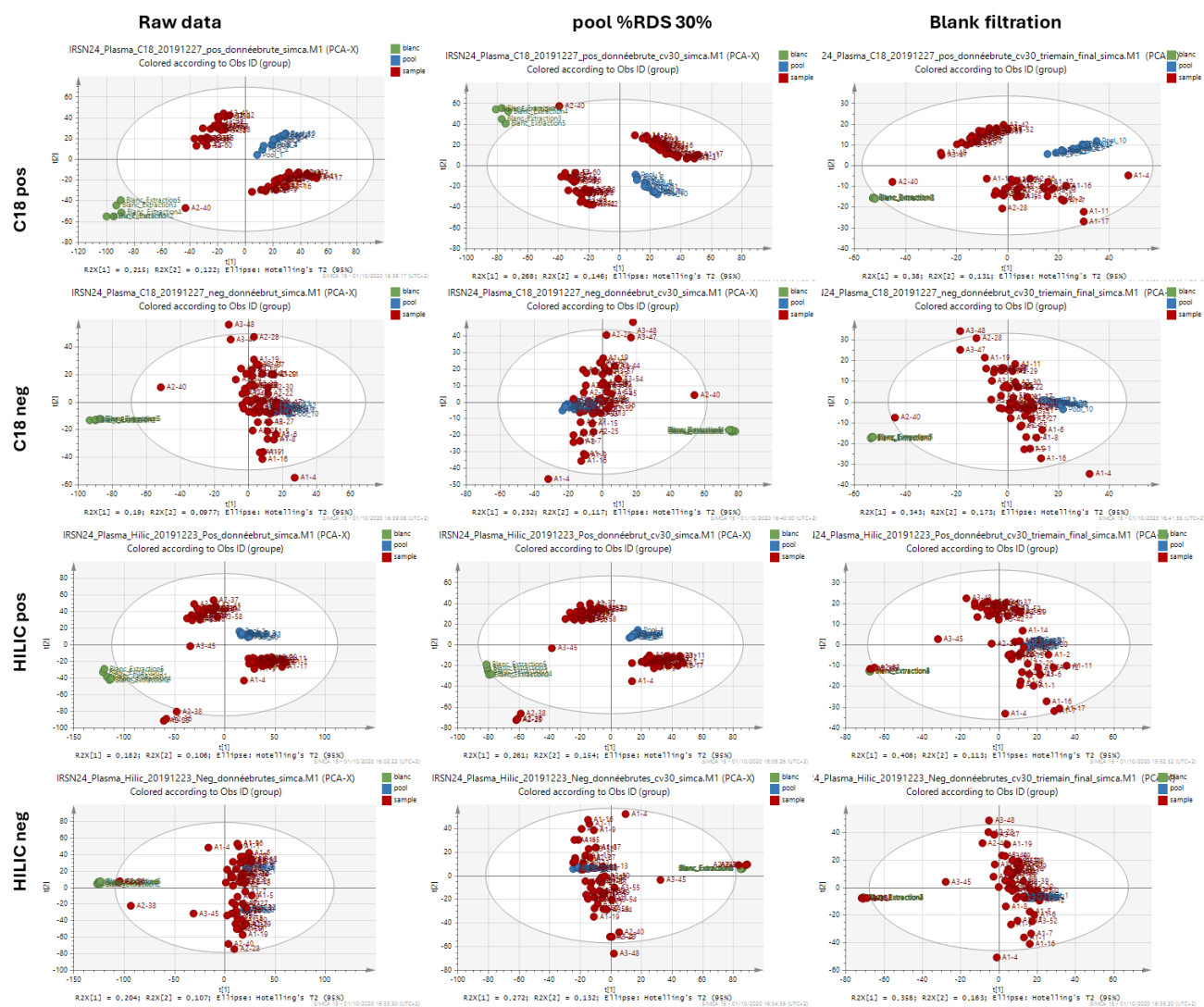

Supplement: Supplementary file 2 — Supplementary Material 2 [file 41598_2025_85717_MOESM2_ESM.pdf]

# Supplementary Figure 2. Enrichment analysis

Overview of Enriched Metabolite Sets (Top 25)

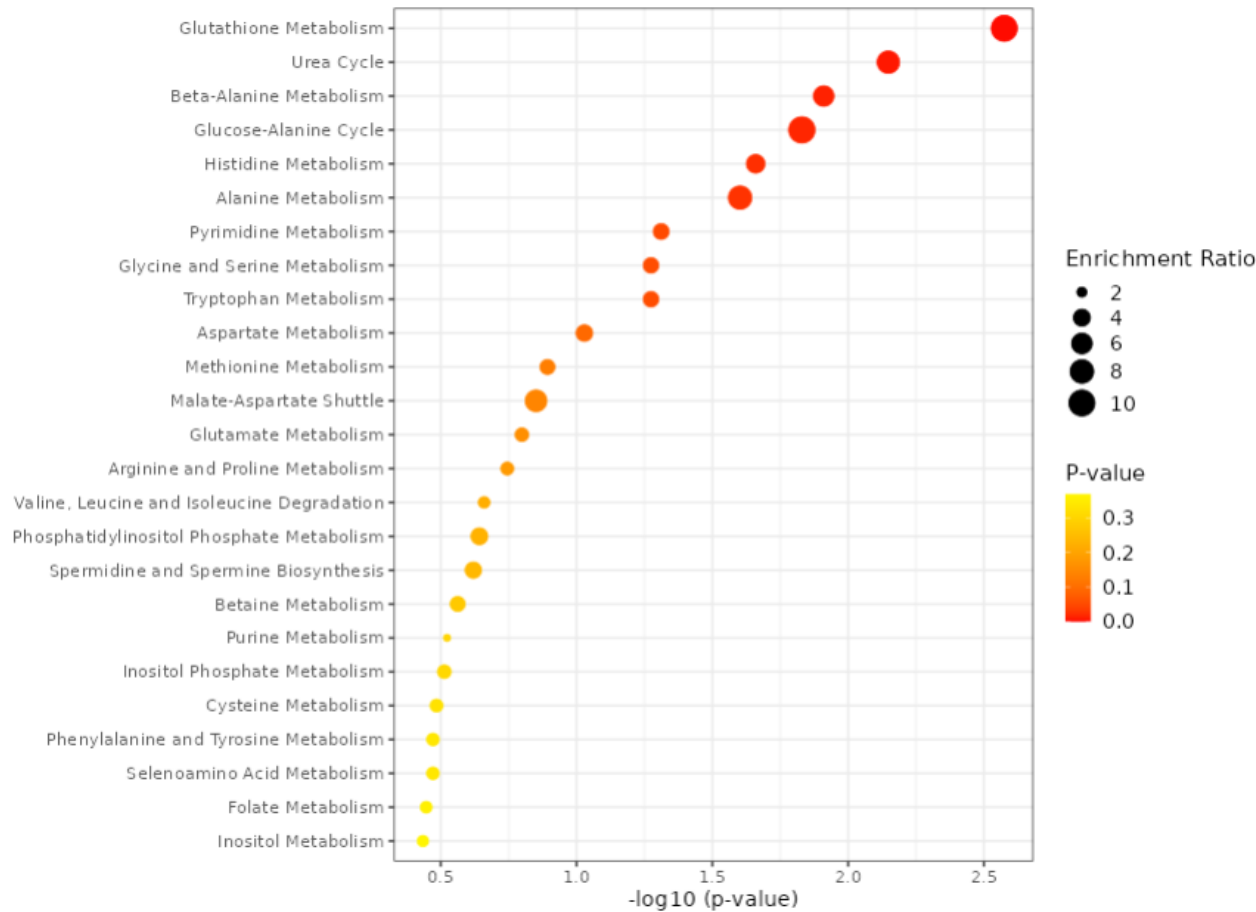

Supplement: Supplementary file 3 — Supplementary Material 3 [file 41598_2025_85717_MOESM3_ESM.pdf]

# Supplementary Figure 3

## Fold Change of 6 metabolites from diagnostic signature

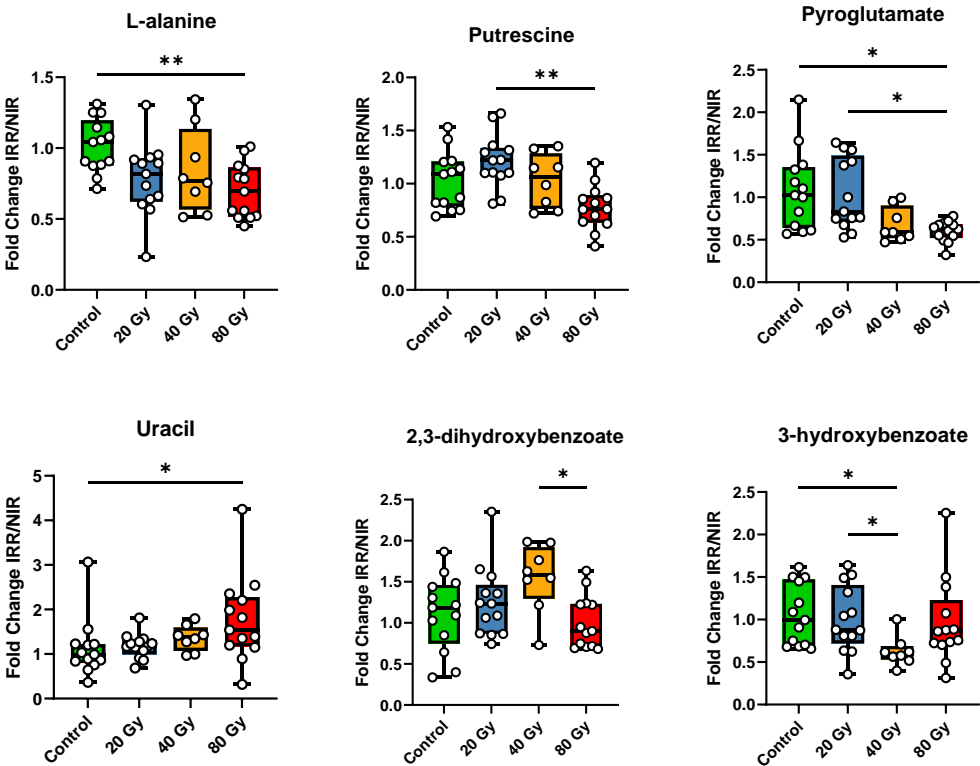

Supplement: Supplementary file 4 — Supplementary Material 4 [file 41598_2025_85717_MOESM4_ESM.pdf]
